# Supplementary material for: AI-assisted optimization design of seismic performance parameters for timber structures
Source: PLoS One. 2026 Apr 8;21(4):e0341961. doi: 10.1371/journal.pone.0341961 (PMC13061333; doi:10.1371/journal.pone.0341961)
Supplement: Attachment — Submitted filename: Review comments.docx [file pone.0341961.s002.docx]

**AI-Assisted Optimization Design of Seismic Performance Parameters for Timber Structures**

We've checked your submission and before we can proceed, we need you to address the following issues:

1.Thank you for updating your data availability statement. You note that your data are available within the Supporting Information files, but no such files have been included with your submission.

At this time we ask that you please upload your minimal data set as a Supporting Information file, or to a public repository such as Figshare or Dryad. Please also ensure that when you upload your file you include separate captions for your supplementary files at the end of your manuscript. As soon as you confirm the location of the data underlying your findings, we will be able to proceed with the review of your submission.

We are requesting these changes to comply with the PLOS ONE submission guidelines ([https://journals.plos.org/plosone/s/submission-guidelines](https://journals.plos.org/plosone/s/submission-guidelines)). Please note that we won't send your manuscript for review until you have resolved the above requests.

Kindly check and update mam

**Response to Editorial Request: Data Availability Clarification**

**Data availability statement**

All data underlying the findings of this study are publicly available without restriction from the Kaggle repository at the following link: [[https://www.kaggle.com/datasets/freshersstaff/timber-seismic-performance-dataset/data](https://www.kaggle.com/datasets/freshersstaff/timber-seismic-performance-dataset/data)]. No additional data are required to reproduce the reported results.

**Supporting Information**

We have now uploaded the minimal dataset underlying the findings of this study as **Supporting Information** with the submission. In addition, the dataset is publicly available without restriction from the Kaggle repository at the following link:

[https://www.kaggle.com/datasets/freshersstaff/timber-seismic-performance-dataset/data](https://www.kaggle.com/datasets/freshersstaff/timber-seismic-performance-dataset/data)
We have updated the Data Availability Statement and added a separate Supporting Information caption at the end of the manuscript in accordance with PLOS ONE submission guidelines. We trust that this resolves the issue and kindly request that the manuscript proceed to peer review.
