# Supplementary material for: AI-assisted optimization design of seismic performance parameters for timber structures
Source: PLoS One. 2026 Apr 8;21(4):e0341961. doi: 10.1371/journal.pone.0341961 (PMC13061333; doi:10.1371/journal.pone.0341961)
Supplement: Attachment — Submitted filename: renamed_fedfb.docx [file pone.0341961.s003.docx]

This paper, titled "AI-assisted optimization design of seismic performance parameters for timber structures", proposes and validates an intelligent framework that integrates gradient boosting random forest and scalable cheetah optimizer (GBRF-SCO) for accurately predicting and optimizing the seismic performance parameters of timber structures. The study shows that this framework has achieved excellent performance on public datasets, providing an effective AI-assisted decision-making tool for seismic design of timber structures.

The author has fully responded to the previous review comments, making important additions and revisions in method details, fairness of comparison experiments, and research limitations, significantly enhancing the rigor and completeness of the manuscript. However, there are still some areas for improvement in the presentation of details, mainly including: units and terms need to be uniformly used throughout (such as the capitalization of Pa and Hz, the spelling of inter-story drift); the clarity of some charts needs to be improved (such as avoiding overlapping elements and enhancing resolution); the reference citation format needs to be uniformly checked (such as the format of author names); and the conclusion part can be further condensed to more focus on highlighting the core innovation, performance advantages, and engineering value of this research.

These issues are of normative or expressive nature and do not affect the core methods and conclusions of the research. It is recommended that the author make revisions based on the following specific suggestions and then consider publication.

1. Ensure consistent use of the International System of Units (SI) throughout the manuscript. For example, in Table 3, "Pa" should be used instead of "pa", and "Hz" instead of "hz". The spelling of "inter-story drift" and "inter-storey drift" must be standardized to a single variant. It is recommended to change all instances of "inter-storey" to "inter-story" in Table 3 and Section 4.6, and to thoroughly check the entire manuscript for this term to ensure uniform usage.

2. The resolution of some charts can be further improved to ensure clear readability after publication. For example, Figures 6, 9, and 15, the contrast and resolution can be appropriately increased to avoid overlapping elements.

3. The author names in the literature are not uniformly formatted, for example, in literature 19 and literature 24, the format of the same name is inconsistent, and it needs to be uniformly verified and checked.

4. The conclusion part has repetitive expressions. It is recommended to focus on the core contributions: the innovative points of GBRF-SCO, the key performance indicators advantages, and the engineering application value, and delete the details that are repetitive with the method section, to enhance the focus of the conclusion.
